# A Cross-Flow Ultrasound-Assisted Extraction of Curcuminoids from *Curcuma longa* L.: Process Design to Avoid Degradation

**DOI:** 10.3390/foods9060743

**Published:** 2020-06-04

**Authors:** Arianna Binello, Giorgio Grillo, Alessandro Barge, Pietro Allegrini, Daniele Ciceri, Giancarlo Cravotto

**Affiliations:** 1 Dipartimento di Scienza e Tecnologia del Farmaco, University of Turin, Via P. Giuria 9, 10125 Turin, Italy; arianna.binello@unito.it (A.B.); giorgio.grillo@unito.it (G.G.); alessandro.barge@unito.it (A.B.); 2 INDENA S.pA., Viale Ortles, 12, 20139 Milan, Italy; pietro.allegrini@indena.com (P.A.); daniele.ciceri@indena.com (D.C.)

**Keywords:** *Curcuma longa* L., curcuminoid stability, multi-step extraction, ultrasound-assisted extraction, extraction kinetic

## Abstract

Rhizomes of *Curcuma longa* L. are well known for their content of curcuminoids, which are compounds with interesting biological activity against various inflammatory states and diseases. Curcuminoids can degrade during processing. This piece of work investigates fast, efficient and cost-effective metabolite recovery from turmeric under ultrasound-assisted extraction (UAE). An analytical evaluation of curcuminoid stability under sonication in different solvents is reported for the first time. HPLC and quantitative ^1^H-NMR were used. Under the applied conditions, EtOAc was found to be the optimal extraction medium, rather than EtOH, due to its lower radical generation, which facilitates better curcuminoid stability. Kinetic characterization, by means of the Peleg equation, was applied for single-step UAE on two different rhizome granulometries. Over a time of 90 min, maximum extraction yields were 25.63% and 47.56% for 6 and 2 mm matrix powders, respectively. However, it was observed that the largest portion of curcuminoid recovery was achieved in the first 30 min. Model outcomes were used as the basis for the design of a suitable multi-step cross-flow approach that supports and emphasizes the disruptive role of cavitation. The maximum curcuminoid yield was achieved over three steps (92.10%) and four steps (80.04%), for lower and higher granulometries, respectively. Finally, the central role of the solvent was further confirmed by turmeric oleoresin purification. The EtOAc extract was purified via crystallization, and a 95% pure curcuminoid product was isolated without any chromatographic procedure. No suitable crystallization was observed for the EtOH extract.

## 1. Introduction

**Figure 1:**
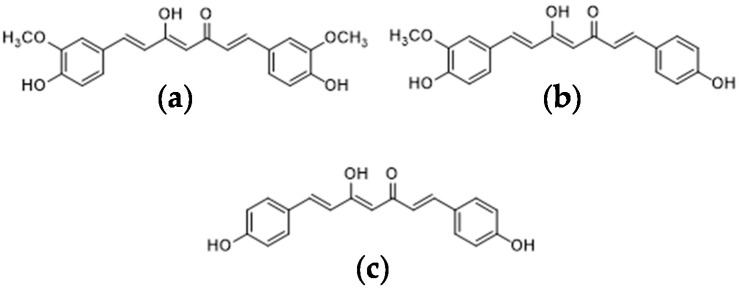
Chemical structures of curcuminoids: (**a**) curcumin (CUR), (**b**) demethoxycurcumin (DMC), (**c**) bisdemethoxycurcumin (BDMC).

In recent years, non-synthetic and biologically active compounds from vegetal sources have gained increasing interest because of their important role in health-care systems worldwide; pigments have found use as additives or supplements in food, pharmaceutical and cosmetic industries. One of the most widely studied sources of natural pigment plants is *Curcuma longa* L., a perennial rhizomatous shrub belonging to the *Zingiberaceae* family. Also known as turmeric, this plant is commonly used as a coloring and flavoring agent in the food industry and, in particular, it is known as the ‘golden spice of life’, and constitutes the main component of curry [1]. However, it is also appreciated in traditional medicine for its biological properties, which are mainly related to its curcuminoids; chemical components that include curcumin (CUR), demethoxycurcumin (DMC) and bisdemethoxycurcumin (BDMC) (Figure 1) [2]. 

Although these substances can be chemically synthesized, is worth noting that the Joint FAO/WHO Expert Committee on Food Additives (JECFA) specifications only allow curcuminoids extracted from natural source material to be used as food additives [3].

Turmeric has curcuminoid contents of 2–9% depending on its growing conditions and origin. The main biological activities exhibited by these compounds are antioxidant, anti-inflammatory, antibacterial, antiviral, antifungal, anticancer, immune-stimulatory and neuroprotective [4,5,6,7].

The most common techniques to obtain curcuminoids from turmeric involve solvent extraction followed by column chromatography. However, the choice of the solvent and the conditions applied must take into account the safety of the final use to which these compounds are intended, for example, their acceptability in the food industry. Soxhlet, ultrasonic-assisted extraction (UAE) and microwave-assisted extraction (MAE) are currently the most commonly used methods, although other techniques, such as pulsed-ultrasonic and supercritical-fluid extraction, are also reported to be efficient processes [8,9,10]. It is worth noting that UAE is now widely used in vegetal-matrix extraction thanks to its efficiency, which allows it to: (1) enhance extraction yields and rates; (2) make use of alternative solvents; (3) reduce costs and required extraction times; and (4) preserve heat-sensitive compounds. Several cavitation devices are available, including bath, probe and flow systems, and can provide huge process-type flexibility, such as scalability, and the use of counter-current or co-current systems [11]. In particular, the co-current cross-flow approach is a relatively simple sequential-step protocol that is used in solid/liquid extraction for different purposes, such as decontamination, leaching and food washing. Essentially, the solid to be extracted is mixed with fresh solvent, then recovered and drained. The extracted solid then meets fresh solvent and is recovered and drained again. The higher the number of steps, the more the matrix approaches depletion [12].

A great deal of metabolite-extraction research has investigated this mechanism, and it has been described using Fick’s second law of diffusion [13,14]. However, a deeper modeling of the kinetics underlying the solid–liquid extraction processes is not common in literature; mathematical assets, such as useful engineering tools, are needed to cast light on method applicability and optimization. Desorption processes, on the other hand, are commonly interpreted by mathematical descriptors in literature, and Peleg’s Model is one of the best known [15]. The common principles shared by dehydration/rehydration phenomena and metabolite extraction allow the ability of this model to render UAE kinetics to be investigated [16,17].

An equation that describes the time-dependent trend of a system can ensure the understanding necessary to define the fastest extraction rates and the best compromises between metabolite yield and time consumption. This approach is fundamental from an industrial point of view, where the main focus is the simultaneous maximization of productivity and the minimization of energy consumption and costs.

The international authorities (FAO/WHO and European Commission), which monitor the development and commercialization of food additives, have decreed that a limited number of solvents are permitted for use in the preparation of curcuminoid-based products [18]. The permitted polar and non-polar organic solvents include acetone, methanol, ethanol, iso-propanol, hexane, ethyl acetate and supercritical carbon dioxide, which have been accepted for use in the extraction of CUR and its analogues from turmeric. 

S.R. Shirsath et al. [17] have thoroughly explored, using kinetic investigations, CUR extraction from *Curcuma amada*, and have screened parameters, such as time, temperature, solvent and granulometry. Ethanol was confirmed as the solvent of choice (vs. methanol, acetone and ethyl acetate), as it was able to give 72% of total metabolites. A direct comparison confirmed the efficiency of UAE, compared to silent conditions which provided an average yield decrease of 10%. A broader screening can be performed to consider other aspects that are involved in the industrial processing of vegetal matrixes, such as easier-to-handle granulometries (less prone to filter clogging), final product stability and purification steps.

In fact, although ethanol is the preferred solvent, the choice of extraction solvent must also be evaluated in relation to the curcuminoid purification steps. On the laboratory scale, chromatography is the technique most commonly employed to isolate curcuminoids, for example using silica as the stationary phase and various organic-solvent mixtures (e.g., chloroform:methanol) as eluents [19]. Crystallization can be included among the purification steps. Ukrainczyk et al. have reported the influence that process conditions have on the purification of crude curcumin via successive cooling crystallizations with isopropanol. [20]. Concentrated oleoresin was selected for the formation of crystals via the slow addition of petroleum ether, water and hexane. The best crystal quality was found when petroleum ether was used, whereas the crystals prepared using the other two solvents were sticky in nature [21]. A non-classical crystallization pathway for curcumin particles was found by Alpana et al. when this process is carried out in sonochemical conditions with or without stabilizers [22].

The quantification of curcuminoid content in turmeric extracts is commonly performed using chromatographic techniques (HPLC, UPLC and capillary electrophoresis). However, in recent years, the quali-quantitative control of herbal products have also been carried out with spectroscopic fingerprinting [23,24]. In particular, Gad and Bouzabata [25] have recently investigated the use of UV, FT-IR and ^1^H NMR for the quality control of *Curcuma longa* by comparing data with those obtained from HPLC analyses. They observed that NMR shows good potential efficiency.

Although curcuminoids possess health-promoting factors, they have found limited application in the food and pharmaceuticals industries because of their low water solubility, poor bioavailability and poor stability in in-vivo and in-vitro environment [26]. These compounds undergo degradation by acidic or alkaline hydrolysis, oxidation, photo-degradation and, moreover, are sensitive to light. The autoxidative degradation of CUR at physiological pH gives bicyclopentandione as the major product, while vanillin, ferulic acid and are reported to be minor compounds [27,28]. The same compounds have been identified as curcuminoid photodegradation products [29].

A systematic stability study of curcuminoids has been carried out by Peram et al. using a RP-HPLC method that analyzes their behavior under different stress degradation conditions (i.e., acidic, alkaline, oxidative, photolytic, and thermal degradation) [30]. The authors reported that the order of stability of curcuminoids was: BDMC, followed by DMC and CUR. This proves that these compounds possess a precise synergistic stabilizing mechanism when present in a mixture, as compared to their pure forms.

The processing conditions that are applied to obtain purified bioactives from turmeric, can affect the labile stability and the biological activity of curcuminoids. Hence, it is of the utmost importance that degradation behavior be considered. Valuable information can be obtained from stability studies in order to understand how the health-promoting effects can be retained. Although mechanistic studies have been published on curcuminoid degradation under different stress conditions [30,31], the kinetics under US treatment, both when curcuminoids are present in standard solutions and when they are found in turmeric extracts, have yet to be reported to the best of our knowledge.

In our work, the rapid and exhaustive UAE of turmeric has been defined using multi step extractions and kinetic studies in ethyl acetate media. Solvent choice was subordinated to bioactive stability and the effectiveness of purification by crystallization.

For the sake of comparison, curcuminoid stability under US irradiation in ethanol and ethyl acetate systems has been tested both on optimized extracts and in standard mixtures.

NMR has been applied as both a qualitative and quantitative analytical method to monitor the stability of curcuminoids under sonication treatment. The degradation products were also detected using HPLC and UPLC-MS analyses.

## 2. Materials and Methods

### 2.1. Chemicals

Ethyl acetate (ACS grade, ≥99%) (Sigma-Aldrich, Milan, Italy) was used in the extraction procedures. Acetonitrile CHROMASOLV^®^ (gradient grade, for HPLC, ≥99.9%) for HPLC analysis was purchased from Sigma-Aldrich, while Milli-Q H_2_O was obtained in the laboratory using a Milli-Q Reference A+System (Merck Millipore). Standards of Curcumin (87.02% Curcumin, 12.98% other curcuminoids) were purchased from Sigma Aldrich.

### 2.2. Curcuma longa L. Matrix

*Curcuma longa* L. mother rhizomes (India), which were cured and sun dried, were kindly provided by Indena SpA (Milano, Italy), in two different average granulometries, 6 and 2 mm. Biomass was ground in a hammer mill. The biomass was stored at room temperature (RT) in a dry and dark environment to avoid metabolite degradation.

### 2.3. Curcuminoid Stability Tests under US

The lability of curcuminoids under US irradiation was evaluated in EtOAc, and compared with the most common GRAS solvent for *Curcuma longa* L. extraction, namely EtOH. In order to evaluate the stabilizing effects of co-extracted molecules, both the dry extract (see conventional extraction, Section 2.5) and the curcumin standard were subjected to sonication prior to solubilization. A total of 2 mL of each solvent were used to dissolve 5 mg of the chosen sample in an analytical tube. The solutions were sonicated in a cup-horn PEX 3 Sonifier (24 kHz, 200W, REUS, Contes, France) for either 30, 60, 90 or 120 min. In order to preserve cavitation efficiency, an average temperature of 40 °C was maintained during the tests thanks to a double layered mantle that was crossed by cooling tap water. After treatment, the samples were dried under vacuum for HPLC analyses.

### 2.4. Ultrasound-Assisted Extraction (UAE)

*Curcuma longa* L. rhizome powder (10 g) was transferred into a 100 mL glass vessel. Ethyl acetate was added, based on a previous screening, to maintain a solid/liquid (S/L) ratio of 1:5. Extraction was performed in two US devices: a probe system equipped with a titanium horn (20.5 kHz, 350-500W, HNG-20500-SP, Hainertec Suzhou, China), and a cup-horn PEX 3 Sonifier (see Section 2.3). The US horn requires an ice bath to control the temperature in the medium. In both systems, an average temperature of 40 °C was maintained to preserve cavitation efficiency while a time screening was performed.

Extractions were conducted in one or multiple sequential steps (cross-flow), with the aim of depleting the matrix.

A technique comparison was performed with 6 mm rhizome powder and the optimized parameters were then transposed to a 2 mm matrix in order to evaluate the incisiveness of mass transport and the physical effects of US treatment. The crude extract was filtered on sintered glass and dried under vacuum.

### 2.5. Conventional Extraction

Classical extractions were carried out as a benchmark for technique screening and for overall curcuminoid yield definition. For the sake of comparison, parameters that were used for optimized UAE, such as temperature, solvent (EtOAc), extraction steps and time, were transposed to the 2 mm matrix in a conventional magnetic stirred system (silent conditions). The maximum curcuminoid yield, which corresponded to matrix depletion, was defined in accordance to the rhizome-powder extraction conditions (S/L ratio 1:5), with homogenization pre-treatment (5 min, OV5 rotor-stator homogenizer Velp Scientifica, Usmate Velat, Italy) and 4 sequential extraction steps (4h each) for a total of 16 h, at RT and under protection from light. For the sake of comparison, the same conditions were used with EtOH as a substitute for EtOAc.

### 2.6. Curcuminoid Determination

Total curcuminoids (TC) were determined by HPLC using the external standard method. Analyses were performed on a Waters 1525 binary pump equipped with a 2998 PDA, and a Phenomenex Kinetex^®^ Column (5 µm C18 100 Å, 250 × 4.6 mm). Data acquisition was accomplished using Empower PRO (Waters Associates, Milford, MA). A CH_3_CN 5% acetic acid aqueous solution was used as the mobile phase. Chromatographic separation was performed in isocratic (50:50 *v*/*v*) at 25 °C, and a flow rate of 1 mL/min. The injection volume was 10 μL, while sample detection was carried out at 425 nm. Before injection, all samples were dissolved in MeOH, giving concentrations of between 1 and 2 mg/mL. All the samples were passed through 0.2-μm membrane filters before injection into the HPLC apparatus. The calibration curve was obtained using curcumin standard solutions (from 0.02 to 2 mg/mL); a linear regression with *R*^2^ > 0.999 was obtained. The reported results express both TC (expressed as curcumin equivalents, percentage yields and mg/mL amounts) and curcumin concentration. Relative limit of detection (LOD) and limit of quantification (LOQ) were determined as 0.005 mg/mL and 0.02 mg/mL, respectively.

### 2.7. NMR Quali and Quantitative Analyses

The quali-quantitative determination of curcumin was also carried out by ^1^H-NMR. NMR spectra were acquired on a Jeol ECZR 600 spectrometer, operating at a 14 T magnetic field strength and equipped with a Jeol Royal standard probe. Signal acquisition and FID processing were carried out using Jeol DELTA software. Qualitative evaluation, via spectrum analysis and a comparison with the spectra of standards, allowed the different curcuminoids that were present in the whole extract to be identified. The quantitation of curcumin was achieved by acquiring ^1^H-NMR spectra in MeOD using a 90° pulse, ^13^C decoupling and a repetition time that was longer than 7-times the longest T1 (typically 40–60 s). FIDs were processed with a zero-filling that was double that of the experimental point (64K+64K), an exponential apodization function with 0.1Hz width and final interactive baseline correction. Specific peak area at 7.20 ppm was compared with the area of potassium terephthalate (analytical standard grade) D_2_O solution (with a precisely known concentration) signals, which were obtained in the same receiver gain conditions as the sample spectra. A set of three different concentrations of standard curcumin was used to verify the correspondence between real curcumin concentration and curcumin concentration as determined by NMR quantitation (using an external standard). Relative LOD and LOQ were calculated as 0.5 mg/mL and 1 mg/mL, respectively.

### 2.8. Statistic Treatment

To validate reproducibility and give soundness to the experimental section, every procedure (Section 2.3, Section 2.4 and Section 2.5) was performed in triplicate and percentual standard deviation was consequently calculated. The results are expressed as the mean ± %SD in Tables as well as in Appendix A. Degradation percentage SDs are graphically depicted in Figures 4–6. Moreover, upper and lower SD envelopes have been extrapolated by means of linear regression, describing deviation trends where possible.

### 2.9. Kinetic Model

The hyperbolic model of Peleg (see Equation (1)) was used to evaluate the extraction kinetics and to determine the point of maximum extraction rate by means of the related constants.

(1) $$C\left(t\right)=C_{0}+\frac{t}{k_{1}+k_{2}t}$$

*C(t)* is the concentration of the extract after extraction time *t*, whilst *C*_0_ is equal to 0 at the beginning of the process. The *Peleg Initial Extraction Rate* (*k*_1_) is correlated to the starting extraction rate (*B*_0_, Equation (2)) and can be exploited to calculate the relative extraction rate in each moment of the extraction (B_t_).

(2) $$B_{0}=\frac{1}{k_{1}}$$

This parameter can be used to calculate the instant of maximum extraction speed, the critical point for cross-flow and counter-current extractions, which is fundamental for an industrial transposition of the process.

The *Peleg Capacity Constant* (*k*_2_) is correlated to the highest extraction yield at the steady state (*Y_s_*, Equation (3)). For an ideal process, it can be used to evaluate the number of sequential extraction steps necessary to deplete the matrix. From a graphical point of view, this parameter represents an horizontal asymptote.

(3) $$c_{0}=c_{eq}=Y_{s}=\frac{1}{k_{2}}$$

Two extraction sets were conducted using a probe system, according to Section 2.4, on the 2 and 6 mm Curcuma powder. Sampling was performed at 2, 5, 7.5, 15, 30, 60, 90 and 120 min, by collecting 1 mL of solution. The crude extract was filtered on sintered glass and dried under vacuum for HPLC analyses.

**Figure A1:**
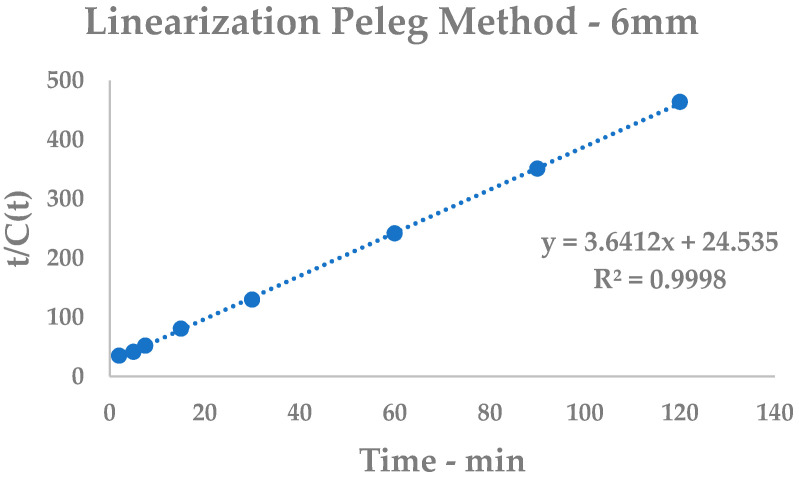
Peleg Model linearization, 6 mm.

**Figure A2:**
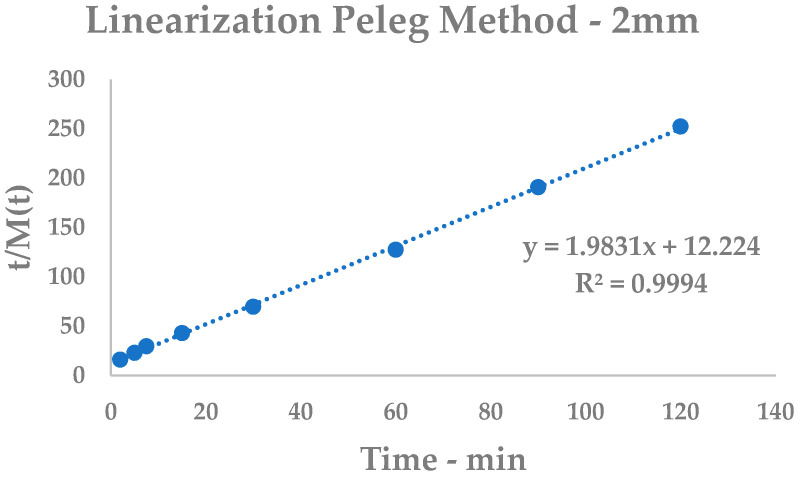
Peleg Model linearization, 2 mm.

Equation (1) can be conveniently linearized in Equation (4), thus providing a fast and easy way to extrapolate *k*_1_ and *k*_2_ as the intercept and slope, respectively. Hence, the kinetic constants can be calculated by linear interpolation of the experimental yields at different extraction times (see Table A1 and Figure A1 for 6 mm and Table A2 and Figure A2 for 2 mm), and then inputted into general Equation (1).

(4) $$\frac{t}{\;C\left(t\right)}=k_{1}+k_{2}t$$

The obtained hyperbolic curve describes a time-dependent extraction trend. This model is a useful means to display the horizontal asymptote of *Y_s_* and the extraction rates (slope of the curve). Furthermore, the knee-point can be exploited to determine the best trade-off between productivity and process extent. 

### 2.10. Crystallization 

The ethyl acetate extracts were concentrated under vacuum to approximately an S/L ratio of 1:1. The mixture was stirred at 20–25 °C for 24 h, then the suspension was filtered under vacuum. The wet solid was ground twice at 20–25 °C for 1 h with 3 volumes of isopropanol 90% (3 mL per g of wet solid). The wet solid was finally dried at 50 °C under vacuum.

## 3. Results

### 3.1. Curcuminoid Stability Test

In order to investigate the effect of the US irradiation of EtOH and EtOAc on curcumin, a known amount of analytical standard was subjected to prolonged sonication in both solvents. Due to the small volume of the samples, the cup-horn was thought to be the most suitable device for the degradation treatments. As shown in Table 1, the effective stability of the pure curcumin was determined by monitoring its concentration every 30 min using HPLC. Total treatment lasted two hours, and the results are reported in Table 1 as curcumin amounts and degradation percentages.

In order to evaluate the degradation that was only related to solvent sonolysis, the degradation test was repeated in EtOAc, in closed vials. The curcumin concentration was determined by quantitative NMR. Although its sensitivity is much lower than HPLC-UV or HPLC-MS, NMR quantitation offers the opportunity to determine the analyte concentration using an external standard whose spectrum is acquired once. The external standard can be a different molecule to the analyte as the only requirement is that its concentration is exactly known, and that its NMR spectrum is acquired following the quantitative protocol. Table 2 reports the comparison between the two behaviors.

Many studies have indicated that curcuminoid stability increases when all of the components are studied together, rather than in their pure form [30]. In order to verify whether this statement can also be applied to US-mediated degradation, the screening that was performed on the analytical standard was repeated on the extract obtained from the conventional procedure (see Section 2.5). The results for EtOH and EtOAc are reported in Table 3.

### 3.2. Total Curcuminoid Content Determination

The quantification of total curcuminoids in plant rhizomes was obtained by performing sequential extractions with EtOAc under ultraturrax^®^ treatment (as described in Section 2.5), with the aim of achieving full matrix depletion. 

The triplicate average gives a total amount of 67.14 mg/g_Matrix_ curcuminoids (HPLC determination, expressed as curcumin equivalents), which corresponds to 6.71% of raw material and 40.49 mg/g_Matrix_ (4.05%) of curcumin. The same extraction protocol was performed with EtOH as the solvent and revealed a negligible difference of +0.84% compared to the previous test. This evidence indicates that no difference subsists between the two solvent systems in terms of total matrix depletion, and that 67.14 mg/g_Matrix_ is admissible as the maximum metabolite yield.

### 3.3. US-Assisted Extraction

This work investigated the UAE of *Curcuma longa* L. and did so by varying US equipment and process parameters. A multi-step approach, which depended on single-step kinetics, was finally defined and investigated for two different granulometries.

Firstly, the effects of acoustic cavitation were studied on the coarse particle size (6 mm), using two different US reactors: an immersion horn (two power intensity, 350 W and 500 W) and a cup-horn (200 W). In order to emphasize the difference between each run, sequential UAE was adopted, and this allowed a preventive evaluation of multi-step feasibility to be performed. Solvent volumes were minimized to an S/L ratio of 1:5, in order to move towards a more sustainable process. Hence, three sequential extractions of 30 min were chosen for each step, in order to avoid the overheating of the system due to solution consistency.

#### 3.3.1. Kinetic Model—Single-Step UAE

Once the extraction device was selected, a better understanding of the kinetic was required. For this purpose, 6 mm rhizomes (treated in Table 4) were considered together with smaller particles (size of 2 mm) for extraction modeling. Samples were gathered after a suitable time-span of 120 min of UAE.

Detected yields were processed according to the Peleg Model, as described in Section 2.9. Using linearization (see Equation (4)), it was possible to extrapolate the kinetic constants by building a general equation for the system that expressed a curve with a physical meaning that describes curcuminoid recovery in a time-dependent function.

**Figure 2:**
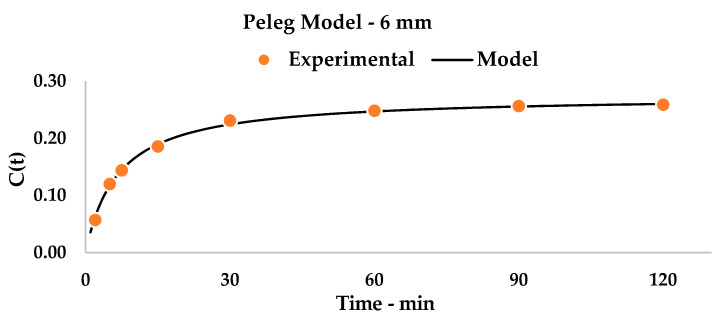
Kinetic Model, 6 mm matrix. Statistical values are reported in Table A1.

**Figure 3:**
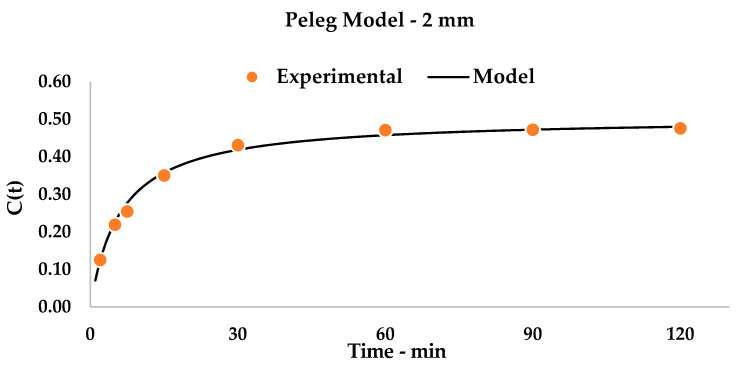
Kinetic Model, 2 mm matrix. Statistical values are reported in Table A2.

The plots (see Figure 2 for 6 mm and Figure 3 for 2 mm) and relative equations (see Equation (5) for 6 mm and Equation (6) for 2 mm) are reported below. Linearization gave impressive interpolation of the experimental values, with R^2^ being over 0.99 (see Figure A1 and Figure A2). It is possible to observe how the theoretical curves match the experimental points.

Extraction rates (B_t_) can be expressed as the inverse of k_1_, which is computable for every instant (t). Bt expresses the extraction efficiency at any precise moment and is mathematically depicted as the slope of a line tangent to the model curve at time t (see Table A1 and Table A2).

(5) $$C\left(t\right)=\frac{t}{24.5350+3.6412\;t}$$

(6) $$C\left(t\right)=\frac{t}{12.2240+1.9831\;t}$$

#### 3.3.2. Cross-Flow UAE

The kinetic model gave 30 min as the best extraction time, as it allowed a good compromise between curcuminoid yield and process duration to be achieved. This discovery was developed and sequential UAE was tested. Biomass was recovered run-by-run and was then submitted to a next stage, feeding fresh EtOAc. Both particle sizes were used in order to define granulometry dependency. Multi-step yields and yield increases are reported in Table 5.

The results depict, as expected, that the extraction rate of the 2 mm rhizome powder was clearly higher than that of the 6 mm analogue. Furthermore, the maximum extraction yield was reached with one extraction step of difference.

### 3.4. Crystallization

The reported procedure (Section 3.1) was applied to both the EtOH and EtOAc extracts. It was found that the alcoholic turmeric oleoresin could not be purified via crystallization, whilst EtOAc successfully led to suitable precipitate formation. HPLC quantification confirmed an average curcuminoid purity of 95%, expressed as curcumin equivalents.

## 4. Discussion

### 4.1. Curcuminoid Stability Test

The fact that radicals are formed as a consequence of acoustic cavitation has been well described in the literature [32]. Although the production of OH^•^ and H^•^ in aqueous media is something that researchers have robustly proven using different approaches [33], very few demonstrations of radical production in organic solvents (as methanol) are available [34]. Radical concentration is frequency-dependent in linear mode, but it is also possible to produce traces of R^•^ or RO^•^ at low frequencies [35]. The extreme reactivity of curcumin-like compounds means that the presence of radicals in the extraction media can affect extraction yields, triggering the degradation and the accumulation of oxidized compounds, which will reduce general shelf-life and product activity.

These considerations can inform the choice of solvent for curcuminoid extractions. Ethanolic solutions are, according to the literature, the best performing media, as they achieve high yields in relatively short times. It is reasonable to think that ethanol is subjected to the formation of CH_3_CH_2_^•^ and OH^•^ radicals, due to his polarity, as these reactive species can also be generated from MeOH and H_2_O [34]. On the other hand, EtOAc (a GRAS approved solvent) is less likely to undergo the same fate.

**Figure 4:**
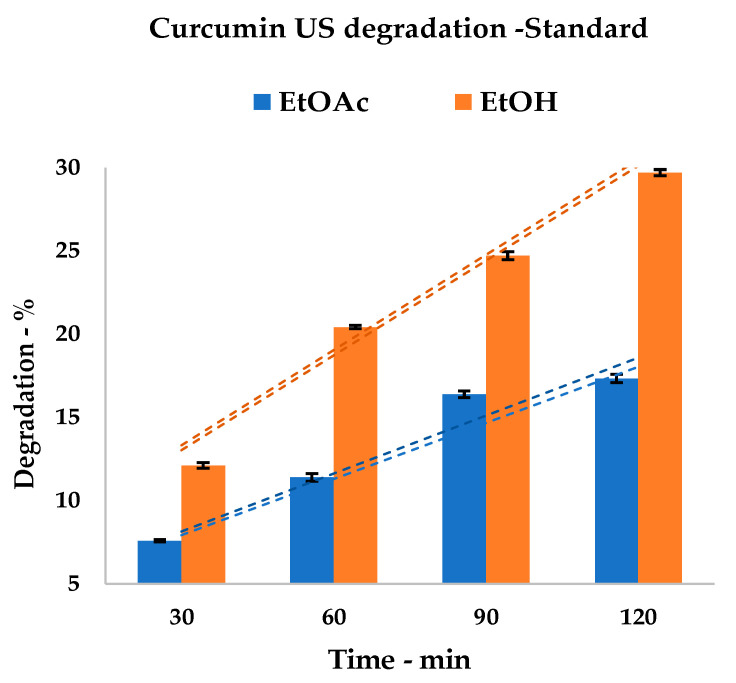
Curcumin standard degradation trend; dashed lines represent upper and lower SD envelopes.

In the case of pure curcumin, the onset of degradation after 30 min supports the low stability of the compound in both solvent systems (Figure 4, related to Table 1). Tests were performed using a common US set-up, in order to be a closer fit for the usual extraction conditions. This approach cannot avoid oxidation due to atmospheric O_2_, which overlaps with the other degradation pathways. In any case, it is possible to state that EtOH media display higher sensitivity than EtOAc media. Furthermore, the time increase leads to a stronger lowering of curcumin concentration in alcoholic systems, reaching a maximum degradation of 29.76% vs. 17.28%.

Tests were repeated in sealed vials to investigate the correlation between atmospheric oxygen and overall degradation. With strongly reduced O_2_ presence, stability should be principally affected by solvent sonolysis.

**Figure 5:**
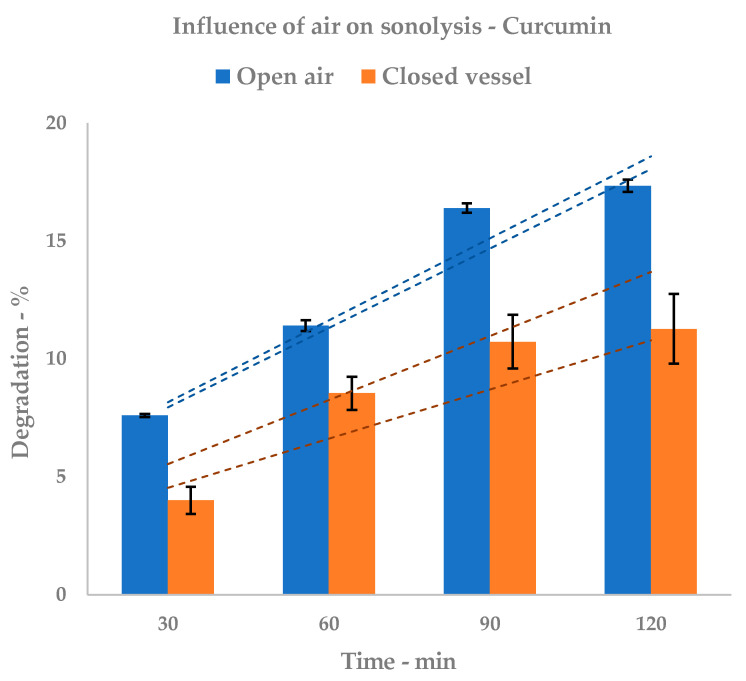
Sonolysis: influence of air. HPLC (open air) and NMR (closed vessel) quantification; dashed lines represent upper and lower SD envelopes.

The concentration of curcumin decreases over sonication time, but its decrease is lower than when measured in open air. The graph depicted in Figure 5 shows behavior over time.

According to the literature, the stability of curcuminoids increases if all three components (CUR, DMC, BDMC) are mixed together [29].

The stability screenings were therefore repeated with the analytical standard of curcumin being replaced with a conventional extract (see procedure in Section 2.5). US extracts were not used as the starting material to ensure that the matrix was not affected by previous irradiation.

**Figure 6:**
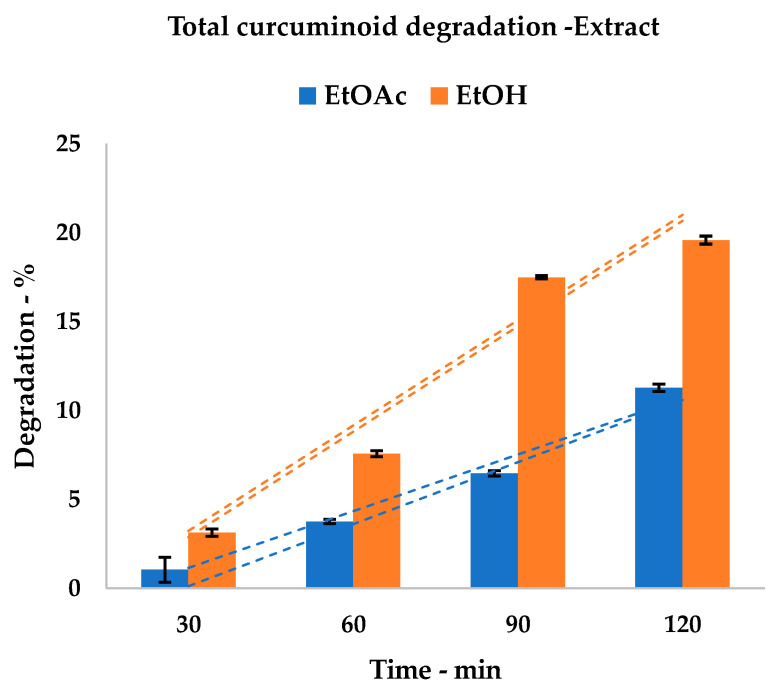
Curcumin extract degradation trend; A: ethanol; B: ethyl acetate; dashed lines represent upper and lower SD envelopes.

The results fit into the abovementioned studies; general stability was increased by the presence of curcuminoids and co-extracts, shifting from 29.76% to 19.56% and from 17.28% to 11.14%, for EtOH and EtOAc media, respectively. It was possible to observe a sudden increase in the degradation of the alcoholic solvent between 60 and 90 min. This is a retardant effect that is possibly due to the presence of the co-extracts (see Figure 6), as this behavior is not visible in pure curcumin (see Figure 4).

These results demonstrate that different solvent media can give rise to different radical concentrations, which is critical for labile molecules, such as curcumin-like compounds, that are well known for their low shelf-life and stability. EtOAc appeared to provide milder radical generation, which paves the way for a suitable multistep extraction protocol that reduces the degradation that is caused by prolonged treatments. These tests are fundamental for a preliminary characterization of a multistep extraction protocol, and for the design of an efficient procedure for the complete depletion of turmeric rhizome powder.

### 4.2. US-Assisted Extraction

In the early stages, the work was focused on 6 mm turmeric powder and finding the best US device for extraction. This approach was a useful means to properly evaluate the physical effects of sonication upon a roughly milled biomass. Bulky powders require better extraction performance for successful cellular/particle cleavage and convenient mass-transfer enhancement to occur. In summary, this type of matrix requires harsher conditions and is a challenge for UAE intensification.

The first approach to UAE was the screening of several US devices, and, in particular, two different set-ups: the immersion horn and cup-horn. The results indicate that a slightly better result was obtained for the horn at 500 W (see Table 4). Technology comparisons shed light on handling and process suitability, and thus influence the practical applications. The cup-horn results were promising even if the power was lower, although temperature control appeared to be very challenging. The sedimentation of heavier particles on the cavitating surface, causing overheated spots, is a possible explanation for this. The media was not easily cooled by the water jacket because of the consequent poorer mass-transfer. The immersion horn, on the other hand, appeared to efficiently transmit energy to the media, despite the radiating surface being smaller. The free tip maintained high mass transfer and easier heat transfer towards the cooling bath. Hence, this first screening facilitated the choice of the 500 W immersion horn as the elective device for the subsequent investigations.

#### 4.2.1. Kinetic Model—Single Step UAE

After the selection of the US device, and before performing the cross-flow extraction design, it was essential to characterize a single-step process. Thus, UAE was approached from a kinetic point of view in order to shed light on the dependency of yield on treatment time. The influence of granulometry was considered for this screening, including the smaller particle size of 2 mm. 

When comparing the two kinetic systems (Figure 2 and Figure 3), some considerations can be pointed out. Firstly, the k_2_ constants describe, as their inverse values, the curcuminoid total yield in the steady state, and are translated by a horizontal asymptote on the graph. This quantity is, as expected, higher for the 2 mm matrix than for the 6 mm one, as it describes, for the first, a matrix that is more prone to extraction (Y_S_: 50.43% vs. 27.46%). Interestingly, the chosen step-length for the initial optimization (30 min, Table 4) appears to give results that are near the maximum yield for single-step extractions: 23.10% vs. 27.46% for 6 mm and 43.10% vs. 50.43% for 2 mm, respectively. This information allowed us to focus our attention on a dedicated multistep cross-flow UAE design.

#### 4.2.2. Cross-Flow UAE

Sequential UAE was based on a value of 30 min as it was the best productivity trade-off for single-step extractions.

**Figure 7:**
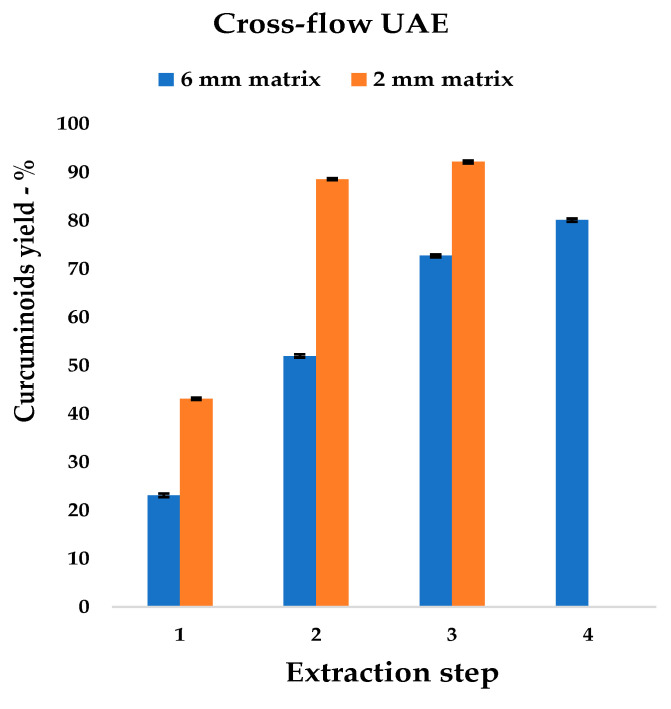
Cross-flow trend for 6 and 2 mm particle sizes.

The highest curcuminoid yield for the 6 mm matrix was obtained with four-step cross-flow, and a progressive reduction in extraction rate was observed (Table 5). As expected, the curcuminoid output steeply increased with the cross-flows and was more pronounced in the 2 mm than in the 6 mm matrix; the yield was almost double at the first extraction (43.10% vs. 23.10%, also see Figure 7).

It is interesting to observe that the maximum production achieved for the coarse material after 4 steps, is overcome by 2 mm turmeric with two UAE steps (80.04% vs. 88.54%, respectively). A fourth extraction was not suitable with the finer Curcuma powder because of the poor quantity of the curcuminoids left in the rhizomes (less than 8%); an additional step was considered not to be cost-effective.

Due to its nature, it is possible to describe a cross-flow protocol (fresh solvent on recycled biomass), as a progressive sum of single extraction stages. Starting from this approximation, experimental yields can be compared step-by-step to the Peleg Model as extrapolated by a reiteration of every stage. In Table 6, it is possible to observe the progress of the model experiments in relation to their step number.

The prediction of sequential extractions using model reiterations clearly shows dramatic discrepancies from the experimental data. This behavior can be explained by the constant biomass modification produced by acoustic cavitation. A likely explanation may be the capacity of US to physically disaggregate vegetal materials [36]. UAE reduces particle size during extraction, enhancing the mass-transfer and modifying the kinetic profile, and thus explains the underestimation of the theoretical approach. 

Moreover, it is possible to state that the observed trend for sequential extraction changes when approaching matrix depletion. This is possibly due to the increase in the magnitude of the degradation mechanisms in light of the accumulation of the treatment periods. Indeed, according to Section 3.1, the total degradation for curcuminoid extracts in EtOAc, is 6.45% after 90 and 11.14% after 120 min (equal to 3 and 4 steps). As a matter of fact, the peculiarities of UAE, such as particle comminution and mass-transfer enhancement, helped to provide a constant yield increase, thus balancing and overcoming curcuminoid degradation.

**Figure 8:**
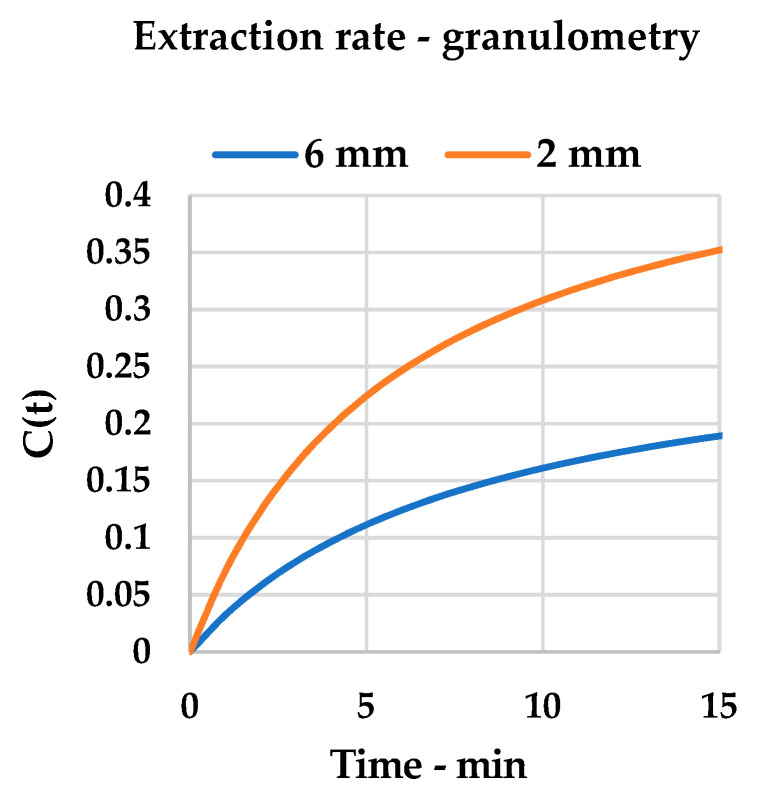
Granulometry comparison: slope variation during initial extraction phase, Peleg model.

The granulometric variation can also be investigated by means of B_t_ values (Equation (2), Table A1 and Table A2). As the most relevant evolution of this parameter occurs in the initial moments of extraction (see Figure 8), the first three points sampled for Peleg’s Model are taken as being descriptive.

**Figure 9:**
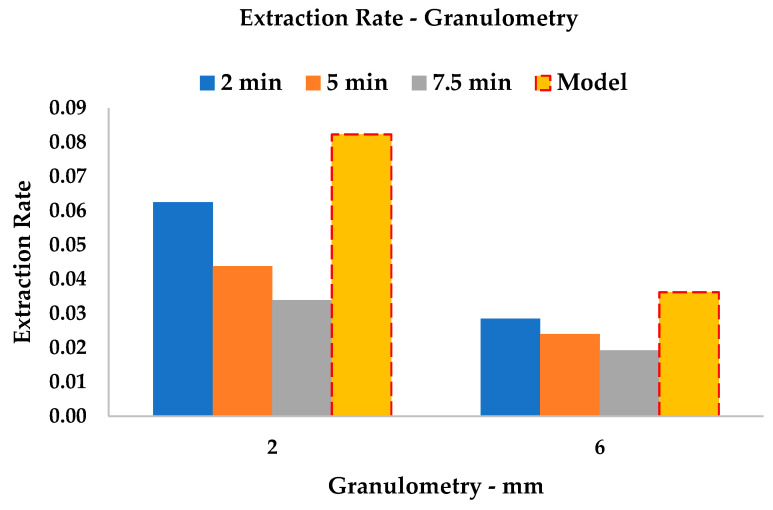
Extraction-rate variation with granulometry. B_t_ of 2, 5 and 7.5 min compared to overall B_0_.

Hence, it is possible to appreciate the slope divergence by plotting the *extraction rates* (Figure 9) for 2, 5 and 7.5 min for every particle size. The reverse proportionality of B_t_ to extraction time was confirmed. It is important to underline that the dashed bars represent the average *extraction rate*, which results from the accumulation of all the rates and is extrapolated from the kinetic constant k_1_, of the systems (B_0_). Therefore, although cavitation can progressively reduce particle size during extraction, the early stages, in which disaggregation is negligible, have a prevailing influence on the overall process.

### 4.3. Crystallization

The features and the advantages of using EtOAc as the extraction solvent were also investigated in terms of metabolite purification via crystallization. In addition to the better stability against curcuminoid radical degradation, the acetate showed an interesting predisposition to crystal generation, which was not observed in the EtOH extracts, under the tested conditions. The efficiency of this purification step is crucial if complex and expensive protocols, such as chromatography, are to be avoided. The final detected purity of the crystallized curcuminoids is 95% on average, as determined by HPLC and expressed as curcumin equivalents.

## 5. Conclusions

A thorough investigation into curcuminoid stability under US interactions has been performed with the elective solvent and its derived ester (EtOH vs. EtOAc). The latter was expected to be less prone to sonolysis. For the sake of comparison, both pure curcumin and a curcuminoid mixture were tested, and HPLC-UV and quantitative ^1^H-NMR were used to determine metabolite degradation. The main advantage of this method is the mere requirement of one analysis for the defined external reference, and no specific and expensive standard for each target analyte. Stability tests have shown that solvents can lead to different radical concentrations, which is crucial for labile molecules, such as curcumin-like compounds. US irradiation caused milder radical generation in the presence of EtOAc than in EtOH, even in relation to the presence of oxygen. In particular, the use of a sealed vessel decreased curcumin degradation by 6.01% after 2 h of US irradiation in the aprotic solvent. The synergistic stabilizing mechanism of curcuminoids when present in a mixed form was confirmed for both the systems, as reported in the literature. In detail, degradation behavior was strongly prevalent in EtOH, with 12.48% and 8.42% decreases being observed for curcumin and the curcuminoids mix, respectively, after 2 h.

These preliminary tests have designated EtOAc as the elective solvent for efficient US-assisted extraction procedures that aim for curcuminoid stability and maximum yields. The use of UAE was first evaluated in a single-step extraction of *Curcuma longa* L., and the impact of different granulometries, namely 6 and 2 mm, was screened. Particular attention was paid to the kinetic features. UAE equilibria were reached before rhizome depletion (25.63% and 47.56% for 6 and 2 mm, respectively), proving the need for a multi-step protocol. Stability tests supported the use of EtOAc, even for sequential extractions, due to its capacity to minimize degradation. Hence, the multi-step co-current cross-flow approach was exploited to maximize yield, and the influence of granulometry is to be highlighted. Maximum curcuminoid recovery was achieved in three steps (92.10%) and four steps (80.04%), respectively, for the 2 and 6 mm rhizome powders.

Comparisons between single extraction kinetics and the cross-flow trend highlight the disaggregation power of US, which is able to accelerate extraction through sequential stages. EtOAc has shown interesting results and applicability in the UAE of *Curcuma longa* L. but also has a crucial role in the final purification steps. In this work, EtOAc produced an extract that is prone to the crystallization process, and an average of 95% pure product was achieved, whilst ethanol prevented crystal formation from the oleoresin extract.

In conclusion, the effect of US on curcumin and curcuminoid degradation has been screened with EtOH and EtOAc for the first time herein, to the best of our knowledge. The results were then exploited to define a UAE process for *Curcuma longa* L. A Peleg kinetic model was used to describe the single-step extraction, and the optimized process time was the groundwork upon which the multi-step cross-flow process was designed. A small particle size was crucial to the obtaining of improved final yields. Finally, the efficacy of the applied solvent was then confirmed by means of suitable curcuminoid purification via crystallization.

## Figures and Tables

**Table 1:** Curcumin standard US degradation tests.

Irradiation (min)	EtOH			EtOAc		
	Curcumin conc. ± %SD (mg/mL)		Degradation (%)	Curcumin conc. ± %SD (mg/mL)		Degradation (%)
0	4.21	± 0.42	-	4.21	± 0.39	-
30	3.70	± 0.61	12.11	3.89	± 0.46	7.60
60	3.35	± 0.54	20.43	3.73	± 0.65	11.40
90	3.17	± 0.73	24.70	3.52	± 0.63	16.39
120	2.96	± 0.68	29.69	3.48	± 0.70	17.34

Curcumin concentration quantified by HPLC.

**Table 2:** Sonolysis: the influence of air on curcumin degradation.

Irradiation (min)	Closed Vessel	
	Degradation	± SD
	(%)	
30	4.00	± 0.58
60	8.54	± 0.72
90	10.72	± 1.14
120	11.27	± 1.48

Solvent: EtOAc; NMR quantifications.

**Table 3:** Curcuminoid extract US degradation tests.

Irradiation	EtOH			EtOAc		
	Curcuminoid conc. ± %SD		Degradation	Curcuminoid conc. ± %SD		Degradation
(min)	(mg/mL)		(%)	(mg/mL)		(%)
0	3.83	± 0.39	-	4.79	± 0.51	-
30	3.71	± 0.61	3.13	4.74	± 0.70	1.05
60	3.54	± 0.57	7.57	4.61	± 0.38	3.76
90	3.16	± 0.49	17.49	4.48	± 0.57	6.47
120	3.08	± 0.67	19.58	4.25	± 0.44	11.27

HPLC quantification of curcuminoids, expressed as curcumin equivalents.

**Table 4:** US technology screening.

US Technology	Dry Extract	Curcuminoid conc. ± %SD	
	(%)	(%)	
Cup-horn ^a^	19.19	72.07	± 0.22
Horn ^b^	16.31	68.43	± 0.38
Horn ^c^	18.17	72.68	± 0.29

Matrix 6 mm, 3 steps of 30 min, S/L ratio 1:5. ^a^ 200W; ^b^ 350W; ^c^ 500W. Curcuminoid yields, calculated as percentage recovery of total curcuminoid-content. HPLC quantification expressed as curcumin equivalents.

**Table 5:** Cross-flow UAE, step screening with 6 and 2 mm matrix.

Step	6 mm Matrix				2 mm Matrix			
	Dry Extract	Curcuminoid Yield ± %SD		Yield Increase	Dry Extract	Curcuminoid Yield ± %SD		Yield Increase
	%	%		%	%	%		%
1	5.44	23.10	± 0.37	-	11.19	43.10	± 0.22	-
2	12.24	51.97	± 0.33	28.87	22.56	88.54	± 0.19	45.44
3	18.17	72.68	± 0.29	20.71	23.77	92.10	± 0.30	3.56
4	20.12	80.04	± 0.31	7.36	-	-	-	-

30 min per step, S/L ratio 1:5, Horn 500 W; Curcuminoid yields, calculated as percentage recovery of total curcuminoid content. HPLC quantification expressed as curcumin equivalents.

**Table 6:** Model fitting to cross-flow screening.

Step	6 mm Curcuminoid Yield		2 mm Curcuminoid Yield	
	Model	Experimental	Model	Experimental
	%	%	%	%
1	22.92	23.10	41.06	43.10
2	40.59	51.97	65.26	88.54
3	54.20	72.68	79.52	92.10
4	64.70	80.04	87.93	-

Thirty min per step, S/L ratio 1:5, Horn 500 W; curcuminoid yields, calculated as the percentage recovery of total curcuminoid content. HPLC quantification expressed as curcumin equivalents.

## Appendix A

**Table A1:** Experimental UAE of 6 mm *Curcuma longa* L.

t	Curcuminoid Yield ± %SD		B_t_^a^	Linearization
min	%			t/C_t_^b^
0	-		-	-
2	0.057	± 0.23	0.0285	35.0877
5	0.12	± 0.21	0.024	41.6667
7.5	0.144	± 0.32	0.0192	52.0833
15	0.1857	± 0.29	0.0124	80.7754
30	0.231	± 0.37	0.0077	129.8701
60	0.2482	± 0.33	0.0041	241.7405
90	0.2563	± 0.24	0.0028	351.1510
120	0.2588	± 0.24	0.0022	463.6785

**^a^** Extraction rates; **^b^** linearization values.

**Table A2:** Experimental UAE of 2 mm *Curcuma longa* L.

t	Curcuminoid Yield ± %SD		B_t_^a^	Linearization
min	%			t/C_t_^b^
0	-		-	-
2	0.1252	± 0.33	0.0625	15.9744
5	0.2188	± 0.25	0.0438	22.8519
7.5	0.2543	± 0.26	0.0339	29.4927
15	0.3501	± 0.30	0.0233	42.8449
30	0.4310	± 0.22	0.0144	69.6056
60	0.4710	± 0.31	0.0079	127.3885
90	0.4718	± 0.28	0.0052	190.7588
120	0.4756	± 0.29	0.004	252.3129

**^a^** Extraction rates; **^b^** linearization values.
